# Ticks feeding on ruminants and humans in Greece

**DOI:** 10.1186/1756-3305-7-S1-O1

**Published:** 2014-04-01

**Authors:** I Chaligiannis, A Papa, S Sotiraki

**Affiliations:** 1 State Veterinary Laboratory of Thrace, Ministry of Rural Development and Food, Komotini 691 00, Greece; 2 Veterinary Research Institute of Thessaloniki, HAO-Demeter (former NAGREF), Thermi 57001, Thessaloniki, Greece; 3 A' Department of Microbiology, Medical School, Aristotle University of Thessaloniki, Thessaloniki, Greece

## 

Ticks are important vectors of disease and transmit an extensive range of viral, bacterial and protozoan pathogens to livestock in a wide variety of habitats. In recent years, diseases such as babesiosis, ehrlichiosis and anaplasmosis have all shown evidence of increased prevalence and distribution in various parts of Europe. However data concerning the prevalence of ticks and tick borne diseases present in livestock and humans in Greece are limited.

In order to fill this gap we performed the current study to define the existence and prevalence of different tick species found in farm animals and humans. As regards livestock, we focused on ruminants (mainly sheep and goats and, in a lesser extent, cattle) since they are the only ones spend time on pastures. A sufficient number of farms all over the country were visited during 2 tick seasons (from March to October), taking different habitats and animal density around Greece into account. Ticks collected from humans originated from infected individuals who visited hospitals.

In total, 2676 ticks were collected from 26 different prefectures (mainland and islands) all over Greece.

From those, 1,883 were coming from sheep (1201) and goats (681) and identified as: *Rhipicephalus sanguineus *1,216 (64.65%); *R. bursa *495 (26.3%); *R. camicasi *12 (0.6%); *R*. *turanicus *70 (3.7%); *Ixodes ricinus *1 (0.05%); *Dermacentor marginatus *47 (2.5%); *Hyalomma marginatum *5 (0.3%); *H. excavatum *2 (0.1%), *H. dromedarii *31 (1.6%) *H. rufipes *2 (0.1%); *H. impeltatum *1 (0.05%); and *Rhipicephalus *nymph 1 (0.05%). More than half (54.3%) of the above were found in an altitude of 0-300 meters, 37.1% in an altitude of 301-800m and 3.2% in an altitude of >800m.

142 ticks originated from cattle and were identified as: *R. sanguineus *15 (10.5%); *R. bursa *6 (4.2%); *R. camicasi *5 (3.5%); *R. turanicus *6 (4.2%); *H. marginatum *24 17%); *H. excavatum *8 (5.6%); *H. dromedarii *67 (47.2%); *H. rufipes *4 (2.8%); *H. impeltatum *1 (0.7%); H. *anatolicum *3 (2.1%) and *H. turanicum *3 (2.1%).

Finally, 701 ticks were coming from humans and identified as: *R. sanguineus *562 (80.17%); *R. bursa *23 (3.28%); *R. turanicus *34 (4.85%); R. *annulatus *5 (0.71%); *H. marginatum *30 (4.28%); *H. excavatum *2 (0.28%); *H. rufipes *11 (1.57%); *Dermacentor marginatus *2 (0.28%); *Ixodes ricinus *6 (0.85%); *I. gibosus *6 (0.85%) and *Rhipicephalus *nymphs 20 (2.85%).

In conclusion, the majority of ticks found in both animal species and humans examined belonged in the *Rhipicephalus sanguineus *group which is the main vector of *Rickettsia conorii*, while *Hyalomma marginatum*, the vectors of CCHF virus, were also present. The above results were more or less anticipated given the climatic conditions of the area, fact that also explains the low prevalence of *Ixodes *spp.

